# Social Disconnectedness and Mental Health Problems During the COVID-19 Pandemic in China: A Moderated Mediation Model

**DOI:** 10.3389/ijph.2022.1604742

**Published:** 2022-06-09

**Authors:** Ruoshan Xiong, Yiwei Xia, Beihai Tian

**Affiliations:** ^1^ Department of Social Work, College of Humanities and Social Sciences, Huazhong Agricultural University, Wuhan, China; ^2^ School of Law, Southwestern University of Finance and Economics, Chengdu, China; ^3^ Department of Sociology, College of Humanities and Social Sciences, Huazhong Agricultural University, Wuhan, China

**Keywords:** COVID-19, social disconnectedness, perceived isolation, COVID-19 related concerns, mental health problems

## Abstract

**Objectives:** This study aimed to examine the mediating effect of perceived isolation and the moderating effect of COVID-19 related concerns in the relationship between social disconnectedness and mental health problems during the COVID-19 pandemic in China.

**Methods:** A cross-sectional online survey of 11,682 Chinese residents were conducted during the COVID-19 outbreak. Conditional process analysis was performed to test the mediating effect of perceived isolation and the moderating effect of COVID-19 related concerns.

**Results:** Social disconnectedness was positively related to mental health problems, and perceived isolation significantly mediated their relationship. COVID-19 related concerns exacerbated the direct link between social disconnectedness and mental health problems as well as the indirect link *via* perceived isolation.

**Conclusion:** Social disconnectedness was a key predictor of mental health problems during the COVID-19 outbreak. The direct and indirect effects of social disconnectedness on mental health problems were stronger for respondents who had more COVID-19 related concerns. Understanding the underlying mechanisms by which social disconnectedness is related to mental health problems has important practical implications for the prevention of mental health problems during the COVID-19 pandemic.

## Introduction

The 2019 novel coronavirus (COVID-19) pandemic, which has been spreading around the world since January 2020, has become a global public health issue. To contain the spread of COVID-19, Chinese authorities have imposed mandatory lockdown policies and social distancing measures, including stay-at-home orders, home quarantines, limited sizes of gatherings and suspensions of social and transportation services. These measures were initially implemented in Wuhan on 23 January 2020 and then another 95 Chinese cities enforced the measures successively [1]. Although such measures have effectively slowed the transmission of COVID-19 on mainland China, they have placed many people at risk for complete social disconnectedness, which posed threats to their psychological well-being [2–4].

Prior studies have shown that social disconnectedness triggers a wide range of mental health problems [5–8]. The negative impact of social disconnectedness on mental health has emerged as a central public health concern during the pandemic. Prior studies have distinguished two forms of isolation: social disconnectedness and perceived isolation. Social disconnectedness, defined as a shortage of social contact with others, is demonstrated by situational factors such as a small size of social network, loss of social interaction, and low levels of involvement in social events and groups [9]. Social disconnectedness is a status of objective social isolation and an inadequacy of social resources. The importance of social connectedness in maintaining mental well-being has been widely documented in empirical research, while social disconnectedness has been found to be associated with a range of mental health problems [7–9]. Previous studies have linked mental health problems to a number of indicators of social disconnectedness, including having a small social network [10], absence of social network diversity [11], disrupted contact with network members [12], weak social group memberships [13] and lower involvement in social activities [14].

Distinct from social disconnectedness, perceived isolation is the subjective sense of a lack of social resources and network functioning [15], which includes feelings of loneliness and a perceived loss of social relations. Despite that social contact with others and participation in social activities declined during the COVID-19 pandemic due to shelter-in-place and stay-at-home orders, many individuals developed closer relationships with family members and friends and perceived relatively stronger social support [16]. Individuals’ object levels of social disconnectedness can be totally irrelevant to their perceptions of social isolation and loneliness. In this regard, it is necessary to investigate both social disconnectedness and perceived isolation as well as their effects on mental health problems. The role of perceived isolation in the formation of mental health problems has also been widely documented in prior research. Theories of perceived isolation contends that as a deeply rooted human characteristic, feeling connected to others is closely linked to bonding, partnership, and conformity behavior that serve an important role in guaranteeing fundamental human needs such as survival, reproduction as well as social functioning [17]. Overwhelming perceptions of loneliness could impair numerous domains of human functioning, which increase the risk for mental health problems. Consistent with this contention, it has been found that people who perceived stronger isolation and loneliness are more likely to suffer higher levels of mental health problems [3, 17, 18]. Research focusing on the mediating role of perceived isolation has found that social disconnectedness leads to perceived isolation, which in turn contributes to the development of psychological problems [18–20].

Despite that people are confronted with similar health risks, individuals tend to be differentially vulnerable to the influence of social disconnectedness, suggesting that it is important to identify the factors that condition the relationship between social disconnectedness and mental health. Individuals’ COVID-19 related concerns such as heightened worry about contracting COVID-19, concerns about own health and general concerns about the COVID-19 crisis have been recognized as risk factors for psychological well-being during the COVID-19 pandemic [21, 22]. Research has found that concerns regarding the pandemic can drive the levels of mental health problems, to the extent that concerns about the pandemic can be more horrible than the pandemic itself [23, 24]. Although COVID-19 related concerns have been identified as a risk factor for psychological well-being, relatively limited research has examined the interplay between social disconnectedness and COVID-19 related concerns in predicting psychological well-being. An online questionnaire survey has shown that concern about the virus exacerbates the link between social isolation and remote work satisfaction [25]. Although this research did not specifically examine mental health problems, it offered a valuable insight into the moderating effect of COVID-19 related concerns on the relationship between social disconnectedness and mental wellbeing.

To date little research has closely examined the relationship between social disconnectedness and psychological wellbeing among Chinese residents during the COVID-19 epidemic. Moreover, the underlying mechanisms by which social disconnectedness is related to mental health problems have not yet thoroughly understood. The current study aims to address these gaps by constructing a moderated mediation model that examines the mediating effect of perceived isolation and the moderating effect of COVID-19 related concerns in the relationship between social disconnectedness and mental health problems among Chinese residents during the COVID-19 outbreak. Based on research reviewed above, we proposed the following hypotheses:

H1: Social disconnectedness is positively related to mental health problems among Chinese residents.
H2: Perceived isolation mediates the relationship between social disconnectedness and mental health problems. Specifically, social disconnectedness leads to perceived isolation, which in turn increases mental health problems.
H3: COVID-19 related concerns condition the direct and indirect link between social disconnectedness and mental health problems through the following three mechanisms:
  H3a: COVID-19 related concerns moderates the direct effect of social disconnectedness on mental health problems;
  H3b: COVID-19 related concerns moderates the effect of social disconnectedness on perceived isolation, and
  H3c: COVID-19 related concerns moderates the effect of perceived isolation on mental health problems.

**FIGURE 1:**
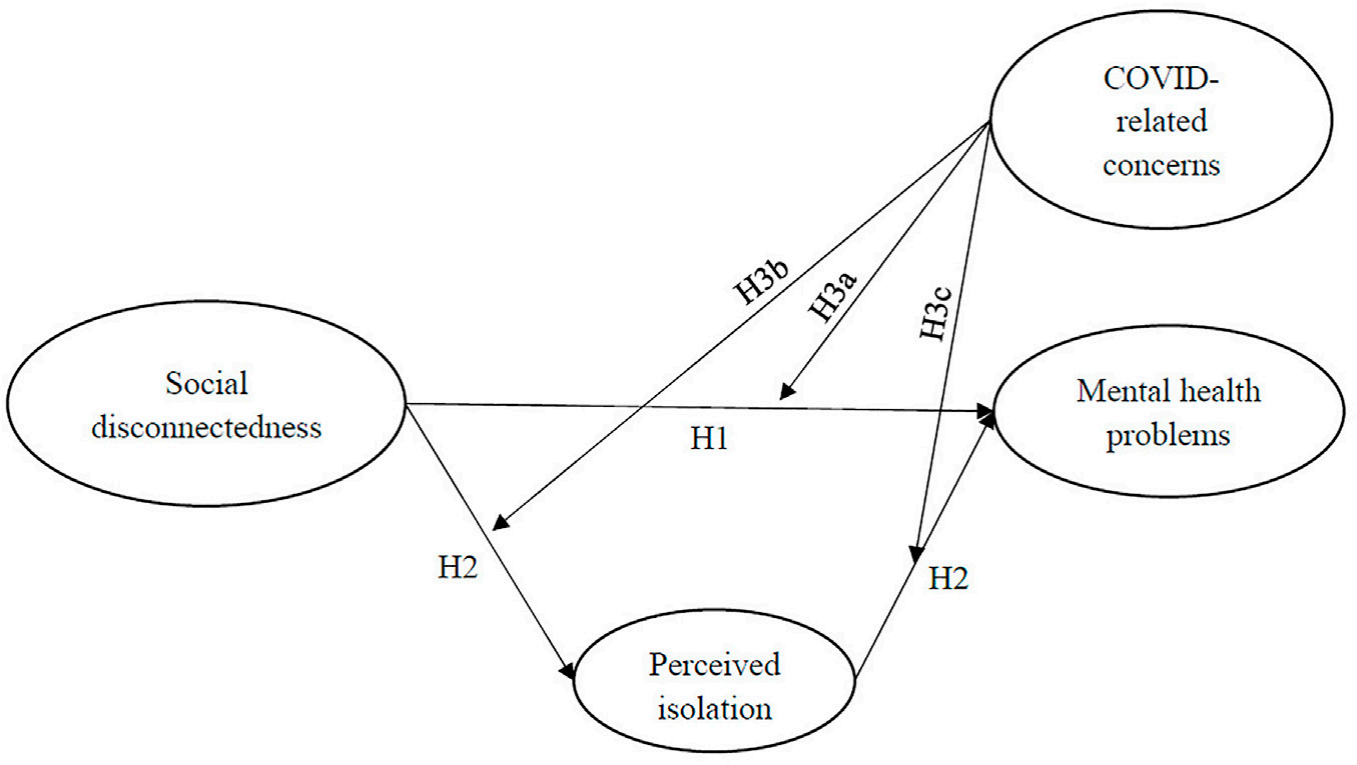
Hypothetical framework. Social Disconnectedness and Mental Health Problems During the COVID-19 Pandemic in China: A Moderated Mediation Model, China, 2020.

The hypothetical framework of this study is illustrated in Figure 1.

## Methods

### Data

The current study used data collected from a large online-based cross-sectional survey conducted from 10 February to 14 February 2020 during the peak period of COVID-19 outbreak in mainland China. Due to concerns of the spread of COVID-19, participants were recruited *via* social media platforms including WeChat and QQ using snowball sampling strategy. All participants completed the self-administered questionnaire online by Ring Survey Platform. The Ethics Committee of the university that funded the project reviewed and approved the survey protocol. Informed consent was obtained from all individual participants included in the study. After excluding samples that have missing values on study variables, a final sample of 11,682 were selected for data analyses.

### Measurement

Social disconnectedness was measured by eight items asking respondents to rate the extent to which they agree each of the eight statements on a 5-point scale ranging from 1 (highly disagree) to 5 (highly agree). Prior research has identified two dimensions, including social network characteristics and social participation to measure social disconnectedness [7, 9]. We constructed 8 items corresponding to the two dimensions to form a scale measuring social disconnectedness. Sample items include: “For now, I have stopped all face-to-face social activities,” “For now, my interactions with my friends and family have decreased” and “For now, I have stopped participating in all group gatherings.” The Cronbach’s value of the eight items was 0.85, indicating a high level of reliability. We used the mean score of the eight items as the measure of social disconnectedness.

Perceived isolation was measured by six items asking respondents to rate the extent to which they agree each of the six statements on a 5-point scale ranging from 1 (highly disagree) to 5 (highly agree). The six items consisted of emotional support from significant others, lack of companionship; feeling lonely; and feeling isolated, which were adapted from previous study of Santini et al. [7]. Sample items include: “Since the outbreak, I have become emotionally distant from my family,” “Since the outbreak, I have become emotionally distant from my friends,” “Since the outbreak, I have often felt a lack of companionship” and “Since the outbreak, I have often felt isolated.” The Cronbach’s value of the six items was 0.90, indicating a high level of reliability. We used the mean score of the six items as the measure of perceived isolation.

COVID-19 related concerns were measured by three items asking respondents the extent to which they are concerned about the COVID-19 pandemic. The three items included general concerns about COVID-19, concerns about contracting COVID-19, concerns about a family member contracting COVID-19, which were consistent with the measurement of this concept in prior research [26]. Each item was rated on a 4-point scale ranging from 1 (not concerned at all) to 4 (very concerned). The Cronbach’s value of the three items was 0.77. We used the mean score of the three items as the measure of COVID-19 related concerns.

Mental health problems were measured by a total of fifteen items, including five questions adopted from Self-Rating Anxiety Scale (SAS) [27], five questions from UCLA Loneliness Scale [28]) and five questions from Geriatric Depression Scale (GDS) [29]. Each respondent was asked to rate the extent to which they agree each of the statement about mental health problems on a 5-point scale ranging from 1 (highly disagree) to 5 (highly agree). Sample items include: “I get upset easily or feel panicky,” “I feel afraid for no reason at all,” “I feel that my situation is hopeless,” “I feel that my life is empty.” The Cronbach’s value of the fifteen items was 0.96. We used the mean score of the fifteen items as the measure of mental health problems.

Control variables included age, gender, education, place of residence, residential type, socioeconomic status and occupation type. Age was an interval variable measured by year, ranging from 14 to 94. Gender (1 = female, 0 = male) were dichotomous variables. Educational level was reported by the respondents on 5 categories from 1 (primary school or less) to 5 (postgraduate or more). Place of residence was measured by asking respondents where you live at present. The response categories are Wuhan, Hubei province (other than Wuhan) and other than Hubei province. Answer categories of the measure of residential type are “1” for “urban area,” “2” for “county,” “3” for “township” and “4” for “rural area.” The respondents rated socioeconomic status on 5 categories from 1 (upper class) to 5 (lower class). Occupation type was a dichotomous variable, with 1 representing high-risk occupations and 0 representing low-risk occupations. High-risk occupations are those with a high risk of contracting COVID-19, including medical workers, community workers, social workers, rescue workers, couriers and drivers.

### Analytical Approach

This study first conducted descriptive analysis to describe the characteristics of the sample. The correlational analysis was then performed to explore the bivariate relationships between the study variables. We then conducted conditional process analysis (CPA) [30] to test the mediating effect of perceived isolation and the moderating effect of COVID-19 related concerns on the link between social disconnectedness and mental health problems. Using OLS regression, we first tested the mediation effect of perceived isolation (model 2 and model 3) and then added the interaction term (model 4 and model 5) to examine the moderating effect of COVID-19 related concerns in the direct and indirect links between social disconnectedness and mental health problems. We estimated model 4 and model 5 using bootstrapping approach with 95% confidence intervals (CI) based on 1000 random samples. The 95% CI does not include zero indicates statistical significance. Data analyses were conducted using Stata 15.1.

## Results

Table 1 presents the descriptive statistics of all the study variables to describe the characteristics of the sample. As shown in the table, the average age of the respondents was 28.98% and 44.49% of the respondents were female. More than half of the respondents (51.89%) received undergraduate education and above (42.00% of them received undergraduate education, and 9.89% received postgraduate or more). In addition, 2.26% of the respondents lived in Wuhan city, 5.55% in other cities in Hubei Province, and 92.19% lived in other provinces. 38.11% of the respondents reported their socioeconomic status was middle and lower class, followed by middle class (33.45%) and lower class (20.94%). About 87.41% of the respondents engaged in low-risk occupations, while 12.59% involved in high-risk occupations. The percentage of respondents living in urban area, county, township and rural area was 35.14%, 28.47%, 13.57%, and 22.82%, respectively.

**TABLE 1:** Descriptive analysis (N = 11,682). Social Disconnectedness and Mental Health Problems During the COVID-19 Pandemic in China: A Moderated Mediation Model, China, 2020.

Variables	Mean/%	Std. Dev	Min	Max
Demographics				
Age	28.98	11.80	14.00	94.00
Gender				
Female	44.49%			
Male	55.51%			
Education				
Primary school or less	3.39%			
Secondary school	23.42%			
Junior college	21.30%			
Undergraduate	42.00%			
Postgraduate or more	9.89%			
Place of residence				
Wuhan	2.26%			
Hubei Province (other than Wuhan)	5.55%			
Other than Hubei Province	92.19%			
Socioeconomic status				
Upper class	1.56%			
Upper-middle class	5.92%			
Middle class	33.45%			
Middle and lower class	38.11%			
Lower class	20.94%			
Occupation type				
High-risk occupations	12.59%			
Low-risk occupations	87.41%			
Residential type				
Urban area	35.14%			
County	28.47%			
Township	13.57%			
Rural area	22.82%			
Independent variable				
Social disconnectedness	3.21	0.54	1.00	5.00
Mediating variable				
Perceived isolation	2.12	1.02	1.00	5.00
Moderating variables				
COVID 19-related concerns	3.21	0.67	1.00	5.00
Dependent variable				
Mental health problems	2.40	1.07	1.00	5.00

The average score of social disconnectedness was 3.21 (out of 5), suggesting the respondents reported experiencing a relatively high level of social disconnectedness. The mean score of perceived isolation was 2.12, indicating that the respondents perceived a moderate level of isolation. The average score of COVID 19-related concerns and mental health problems were 3.21 and 2.40, respectively. The results suggested that the respondents showed at least a certain degree of concerns about the COVID-19 pandemic and experienced a relatively moderate level of mental health problems.

Table 2 shows the pairwise Pearson correlations between the key study variables. The key variables were significantly correlated with each other. As shown in the table, social disconnectedness was positively correlated with perceived isolation (*r* = 0.21, *p* < 0.001) and mental health problems (*r* = 0.24, *p* < 0.001). Table 2 also shows that social disconnectedness was positively correlated with COVID 19-related concerns (*r* = 0.09, *p* < 0.001). Further, perceived isolation was positively correlated with COVID 19-related concerns (*r* = 0.12, *p* < 0.001) and mental health problems (*r* = 0.77, *p* < 0.001). Finally, COVID 19-related concerns was positively correlated with mental health problems (*r* = 0.21, *p* < 0.001).

**TABLE 2:** Zero-order correlation between key variables (N = 11,682). Social Disconnectedness and Mental Health Problems During the COVID-19 Pandemic in China: A Moderated Mediation Model, China, 2020.

		(1)	(2)	(3)	(4)
(1)	Social disconnectedness	1.00			
(2)	Perceived isolation	0.21***	1.00		
(3)	COVID 19-related concerns	0.09***	0.12***	1.00	
(4)	Mental health problems	0.24***	0.77***	0.21***	1.00

All correlation coefficients are standardized.

***p < 0.001.

Table 3 exhibits the standardized coefficients of the CPA analysis and goodness of fit of five models. Model 1 to model 3 in Table 3 revealed that perceived isolation significantly mediated the relationship between social disconnectedness and mental health problems. According to model 1 in Table 3, the direct effect of social disconnectedness on mental health problems was significant (*β* = 0.48, *p* < 0.001), which supported H1. As indicated in model 2, the effect of social disconnectedness on perceived isolation was significant (*β* = 0.43, *p* < 0.001), and the effect of social disconnectedness on mental health problems still reached a level of statistical significance (*β =* 0.15, *p* < 0.001) after controlling perceived isolation in model 3, which lent support to H2. It should be noted that after controlling for perceived isolation, the coefficient of social disconnectedness changed from 0.48 to 0.15, suggesting the mediating effect accounted for 68.75% ((0.48−0.15)/0.48 = 68.75%) of the total effect of social disconnectedness on mental health problems. Thus, there is strong evidence supporting the mediating effect of perceived isolation on the relationship between social disconnectedness and mental health problems (H2).

**TABLE 3:** The results of conditional process analysis (N = 11,682). Social Disconnectedness and Mental Health Problems During the COVID-19 Pandemic in China: A Moderated Mediation Model, China, 2020.

	Model 1	Model 2	Model 3	Model 4	Model 5
	DV: Mental health problems	DV: Perceived isolation	DV: Mental health problems	DV: Perceived isolation	DV: Mental health problems
Age	−0.00***	−0.01***	0.00	−0.01***	0.00*
	(0.00)	(0.00)	(0.00)	(0.00)	(0.00)
Female	−0.11***	−0.22***	0.06***	−0.22***	0.06^***^
	(0.02)	(0.02)	(0.01)	(0.02)	(0.01)
Primary or less	Ref.	Ref.	Ref.	Ref.	Ref.
Secondary school	0.14*	−0.01	0.15***	−0.03	0.13***
	(0.06)	(0.06)	(0.04)	(0.06)	(0.04)
Junior college	0.06	−0.07	0.12**	−0.08	0.11**
	(0.06)	(0.06)	(0.04)	(0.06)	(0.04)
Undergraduate	0.04	−0.10	0.12**	−0.09	0.12**
	(0.06)	(0.06)	(0.04)	(0.06)	(0.04)
Postgraduate or more	0.04	−0.12	0.13**	−0.11	0.15***
	(0.07)	(0.06)	(0.04)	(0.06)	(0.04)
Wuhan	Ref.	Ref.	Ref.	Ref.	Ref.
Hubei Province	−0.24**	−0.18*	−0.10*	−0.18*	−0.10*
	(0.08)	(0.07)	(0.05)	(0.07)	(0.05)
Other Provinces	−0.32***	−0.17**	−0.18***	−0.15*	−0.17***
	(0.06)	(0.06)	(0.04)	(0.06)	(0.04)
Occupation type	0.26***	0.30***	0.03	0.29***	0.02
	(0.03)	(0.03)	(0.02)	(0.03)	(0.02)
Urban area	Ref.	Ref.	Ref.	Ref.	Ref.
County	−0.07**	−0.01	−0.06***	−0.01	−0.05***
	(0.02)	(0.02)	(0.02)	(0.02)	(0.02)
Township	−0.14***	−0.09**	−0.07**	−0.09**	−0.06**
	(0.03)	(0.03)	(0.02)	(0.03)	(0.02)
Rural area	−0.15***	−0.10***	−0.06***	−0.10***	−0.06***
	(0.03)	(0.03)	(0.02)	(0.03)	(0.02)
Socioeconomic status	0.07***	0.09***	−0.01	0.09***	−0.01
	(0.01)	(0.01)	(0.01)	(0.01)	(0.01)
Social disconnectedness	0.48***	0.43***	0.15***	0.26***	0.01
	(0.02)	(0.02)	(0.01)	(0.08)	(0.05)
COVID-19 related concerns				0.00	0.10
				(0.08)	(0.05)
Disconnectedness×concerns				0.05*	0.04*
				(0.02)	(0.02)
Perceived isolation			0.79***		0.85***
			(0.01)		(0.03)
Isolation×concerns					−0.02* (0.01)
Constant	1.19***	1.10***	0.32***	1.11***	0.04
	(0.12)	(0.11)	(0.08)	(0.26)	(0.18)
*R* ^2^	0.08	0.08	0.60	0.09	0.61
adj. *R* ^2^	0.076	0.079	0.599	0.089	0.611
*AIC*	33743.09	32707.64	24000.20	32588.36	23643.30
*BIC*	33853.58	32818.13	24118.05	32713.58	23783.25
F	69.96	72.62	1163.68	71.94	1020.52

Ref. means the reference group.

Standard errors are shown in parentheses.

*p < 0.05, **p < 0.01, ***p < 0.001.

**FIGURE 2:**
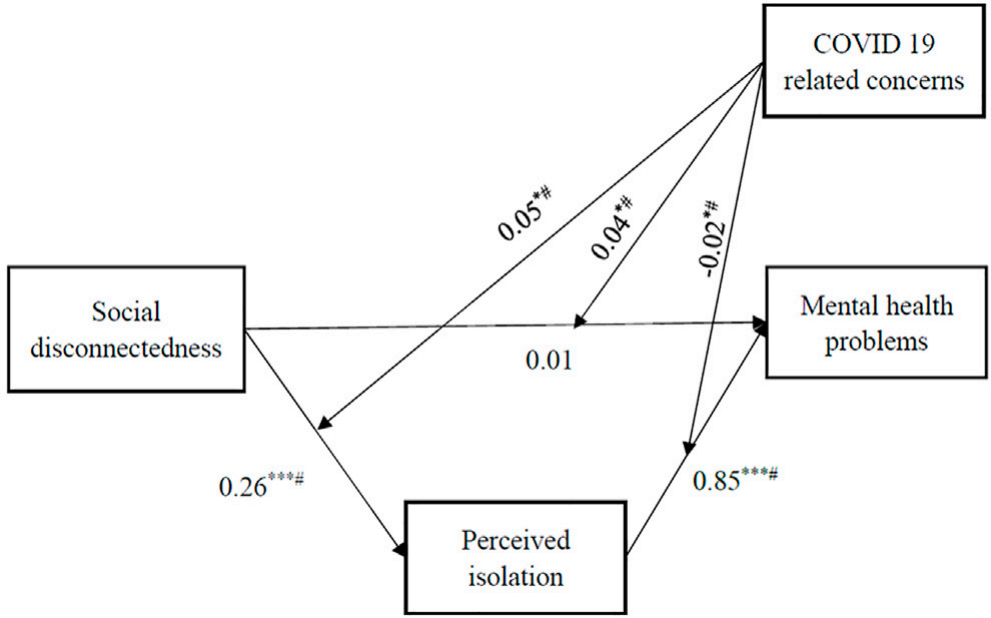
The results of conditional process analysis. Social Disconnectedness and Mental Health Problems During the COVID-19 Pandemic in China: A Moderated Mediation Model, China, 2020. Note: All the coefficients are standardized. Age, gender, education, place of residence, residential type, socioeconomic status and occupation type are controlled for all the endogenous variables. ^#^ indicates bootstrapping confidence interval with 1000 replicates does not include zero. ^*^ *p* < 0.05, ^**^ *p* < 0.01, ^***^ *p* < 0.001.

Model 4 and model 5 added the interaction term (social disconnectedness × COVID-19 related concerns, and perceived isolation × COVID-19 related concerns). Model 4 and model 5 showed that COVID-19 related concerns significantly moderated the link between social disconnectedness and mental health problems through its impact on perceived isolation. As shown in model 4 and model 5 of Table 3, the coefficients of the interaction terms all reached a level of statistical significance and the 95% CIs of statistically significant results do not include zero. Therefore, the results in Table 3 were robust against the bootstrapping approach, supporting H3a, H3b and H3c (*β =* 0.05, *p* < 0.05; *β =* 0.04, *p* < 0.05; *β = -*0.02, *p* < 0.05). Figure 2 shows the standardized coefficients of the CPA analysis.

**FIGURE 3:**
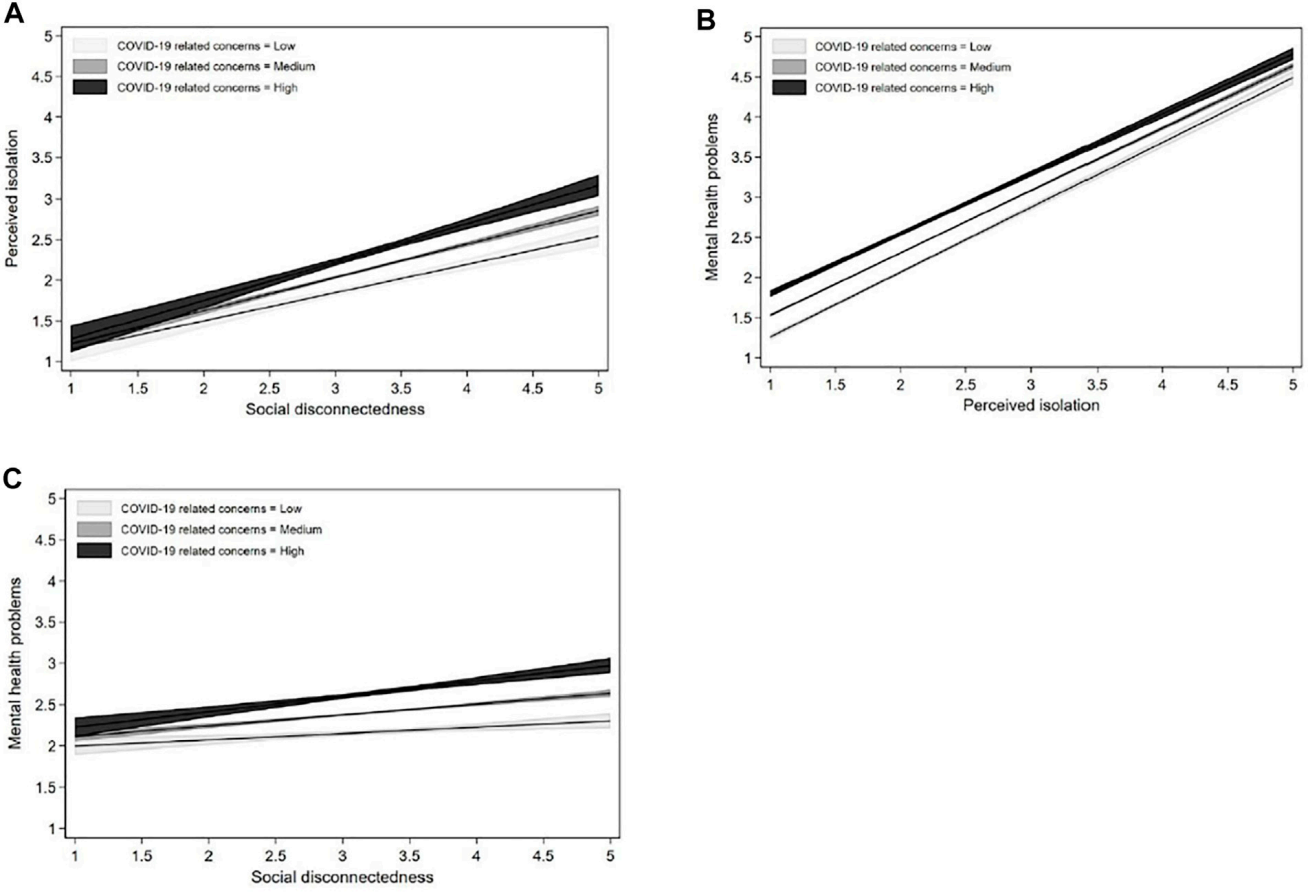
The moderating effect of COVID-19 related concerns. **(A)** The moderating effect of COVID-19 related concerns on the relationship between social disconnectedness and perceived isolation. **(B)** The moderating effect of COVID-19 related concerns on the relationship between perceived isolation and mental health problems. **(C)** The moderation effect of COVID-19 related concerns on the relationship between social disconnectedness and mental health problems. Social Disconnectedness and Mental Health Problems During the COVID-19 Pandemic in China: A Moderated Mediation Model, China, 2020.

To better interpret how COVID-19 related concerns moderate the relationship between social disconnectedness and mental health problems, Figure 3 plots the estimated perceived isolation and mental health problems at different levels of COVID-19 related concerns using the coefficients in model 4 and model 5 in Table 3. The low, medium and high levels of COVID-19 related concerns were defined by the mean minus one standard deviation, the mean, and the mean plus one standard deviation of the measure of COVID 19 related concerns, respectively. Figure 3A simulated a set of values from 1 to 5 by 0.5 to represent social disconnectedness and its CI, and then calculated the corresponding value of perceived isolation based on the results of model 4 in Table 3. As shown in Figure 3A, despite social disconnectedness was positively associated with perceived isolation at all levels of COVID-19 related concerns, the slope became steeper when respondents showed higher levels of COVID-19 related concerns, suggesting that COVID-19 related concerns exacerbated the effect of social disconnectedness on perceived isolation. In the same vein, Figures 3B,C predicts the effect of perceived isolation and social disconnectedness on mental health problems at different levels of COVID-19 related concerns using the results of Model 5 in Table 3. As shown in Figure 3B, although the slope descended slightly when COVID-19 related concerns changed from low to high, respondents who had higher levels of concerns were at a higher risk of developing mental health problems at all values of perceived isolation. For Figure 3C, the slope ascended when COVID-19 related concerns changed from low to high, suggesting that respondents experiencing social disconnectedness were more likely to develop mental health problems when they had a higher level of COVID-19 related concerns. These findings revealed that COVID-19 related concerns significantly exacerbated the relationship between social disconnectedness and mental health problems through its impact on perceived isolation.

## Discussion

The negative impact of social disconnectedness on mental health has emerged as a central public health concern during the COVID-19 pandemic [7, 8]. However, few studies have investigated the relationship between social disconnectedness and mental health and the mechanisms underlying the relationship. To the best of our knowledge, this study is the first attempt to explore the mediating effect of perceived isolation and the moderating effect of COVID-19 related concerns in the relationship between social disconnectedness and mental health problems in the context of the COVID-19 pandemic. Although the COVID-19 epidemic has entered into the remission stage in some countries, the impact of the social disconnectedness on mental health is still a major public health issue, especially for some cities that are still suffering from restricted social contact and participation in social activities due to the repeated outbreak of the COVID-19 pandemic. Moreover, previous studies showed that mental health problems in the general population were higher at the remission than at the outbreak stage [31]. Therefore, this study still plays a role in the current epidemic. By examining the underlying mechanisms underlying the link between social disconnectedness and mental health problems, this study provides a valuable insight about the prevention and intervention of mental health problems and the improvement of people’s psychological well-being in the context of social disconnectedness during the pandemic. The strengths of this research lie in the large sample size collected from a nation-wide survey and the use of verified instrument to measure mental health problems.

This study confirms the first hypothesis by demonstrating that social disconnectedness was positively related to mental health problems, which is consistent with prior research that found a close relationship between social disconnectedness and psychological well-being [4, 32]. The results suggested that social disconnectedness resulting from social distancing measures increased the risk for mental health problems during the peak period of COVID-19 outbreak in mainland China. In line with previous studies [8, 9, 17–20], the mediation analyses found that perceived isolation significantly mediated the relationship between social disconnectedness and mental health problems, which supported the second hypothesis. Social disconnectedness indirectly gave rise to mental health problems by increasing perceived isolation. While prior literature widely documented that the co-occurrence of social disconnectedness and perceived isolation has an impact on mental health [33–35], our study suggested that both social disconnectedness and perceived isolation led to mental health problems, with the former playing a more distal role and the latter a more proximate role in predicting the problems.

In addition, the findings lend strong support for the third hypotheses of the moderating effects of COVID-19 related concerns. In support of the contention that concerns about the pandemic when combined with the absence of social contacts can strongly affect mental health problems [23–25], the conditional process analysis in this study revealed that COVID-19 related concerns aggravated the adverse impact of social disconnectedness and mental health problems. Our findings showed that COVID-19 related concerns achieved its moderating role in three ways. First, COVID-19 related concerns exacerbate the direct effect of social disconnectedness on mental health problems. Second, COVID-19 related concerns aggravate the link between social disconnectedness and perceived isolation. Third, COVID-19 related concerns exacerbate the relationship between perceived isolation and mental health problems. Increased concerns about the pandemic are strong emotions that increase intolerance of uncertainty, affect individuals’ moods and further accentuated the situation [36]. The moderating role of COVID-19 related concerns found in this study contributes to the current knowledge by disclosing the interplay between social disconnectedness and COVID-19 related concerns in predicting mental health problems.

Understanding the underlying mechanisms by which social disconnectedness is related to mental health problems has important practical implications for the prevention and intervention of mental health problems in the face of the pandemic. Interventions adopted to foster social connectedness and mental wellbeing should focus on the underlying mechanisms of change [37]. First, considering perceived isolation represents a proximal risk factor of mental health problems, efforts adopted to improve social connectedness should focus on reducing perceived isolation and enhancing social support [7]. Despite that social connectedness has been interrupted by social distancing measures, people could achieve distanced connectivity to alleviate the risk of perceived social isolation. It is important to build social support system in the time of COVID-19. For example, active measures should be taken to make a range of social support resources available to individuals suffering from social disconnectedness in order to reduce the negative impact of the pandemic on psychological well-being [38]. People also should facilitate connectedness among family members and friends, increase opportunities for social interactions, since family support could reduce perceived isolation and increase the level of life satisfaction during the pandemic [16]. Secondly, our results showed that COVID-19 related concerns aggravated the relationship between social disconnectedness and mental health problems, suggesting that special attention should be paid to alleviate unnecessary concerns about the pandemic. For example, special healthcare center could be launched to answer people’s queries and provide people with verified advice and information to help reduce concerns and promote psychological resilience during the pandemic.

Despite the strengths mentioned in this study, certain limitations should be acknowledged when interpreting the results. First, this study was limited by reliance on self-reported data, which might lead to the possibility for self-report bias, especially for the measure of mental health problems. The results might differ if mental health problems had been measured by clinical diagnosis. Second, the use of cross-sectional data in this study restricted our ability to make a causal interpretation of the results. Third, due to the online nature of the study, the results might be subject to selection bias and likely systematically preclude respondents who are less technologically literate. Therefore, our data probably over-represented urban residents who have wider access to technology, and underrepresented low-income, rural residents and older people who have limited ability to access technology, which might undermine the accuracy of the findings. To address these limitations, future research should use longitudinal data to identify the direction of the relationships between the key study variables and sort out how change in social disconnectedness and perceived isolation is related to mental health problems over time. Additionally, further studies should consider administrating the survey using various means, such as phone and post, to capture the experience of older people and those who have limited access to technology.
